# Impaired P50 sensory gating and depressive symptoms in adolescents with non-suicidal self-injury

**DOI:** 10.3389/fpsyt.2026.1812184

**Published:** 2026-03-25

**Authors:** Shen Li, Yuxin Han, Kangjie Yue, Yue Li, Xiaochun Sun, Lijun Wang, Wenjie Sun

**Affiliations:** 1 Brain Assessment & Intervention Laboratory, Tianjin Anding Hospital, Mental Health Center of Tianjin University, Tianjin, China; 2 Institute of Mental Health, Tianjin Anding Hospital, Mental Health Center of Tianjin Medical University, Tianjin, China; 3 Institute of Mental Health, Tianjin Anding Hospital, Mental Health Center of Tianjin University, Tianjin, China

**Keywords:** adolescents, cognitive function, depressive symptoms, event-related potentials, non-suicidal self-injury, P50 sensory gating

## Abstract

**Background:**

Non-suicidal self-injury (NSSI) in adolescents is highly prevalent and frequently co-occurs with depressive symptoms, yet the neurophysiological mechanisms underlying this comorbidity remain unclear. P50 sensory gating reflects early inhibitory control of redundant sensory input and has been implicated in affective disorders. This study examined whether P50 gating deficits are present in adolescents with NSSI and whether they are associated with depressive symptom severity and cognitive performance.

**Methods:**

Eighty-six adolescents with NSSI (55 with depressive symptoms; 31 without) and 50 healthy controls were recruited. Depressive symptoms were assessed using the 17-item Hamilton Depression Rating Scale (HAMD-17), and NSSI participants were stratified into depression (HAMD-17 ≥ 8) and non-depression (HAMD-17 < 8) subgroups. Cognitive function was evaluated using the MATRICS Consensus Cognitive Battery (MCCB). P50 sensory gating was measured using an auditory paired-click ERP paradigm, with gating indices calculated as the S2/S1 amplitude ratio and S1-S2 difference. Group differences in P50 indices were examined, and correlations and regression analyses were conducted to assess associations between P50 parameters, depressive symptoms, and cognition.

**Results:**

Significant group differences were observed in S2 amplitude and S2/S1 ratio both *P*s < 0.05). Adolescents in the NSSI with depression subgroup exhibited significantly higher S2 amplitude and S2/S1 ratio compared with both healthy controls (S2/S1: *F* = 8.688, *P* = 0.004) and NSSI without depression. After controlling for age and sex, the between-subgroup difference in S2 amplitude remained significant (F = 5.279, *P* = 0.024). Within the overall NSSI sample, S2 amplitude was independently predicted HAMD-17 scores (*β* = 0.264, *P* = 0.009). In the NSSI with depression subgroup, S2 amplitude was negatively correlated with Maze task performance (*r* = -0.298, *P* = 0.027).

**Conclusion:**

P50 sensory gating deficits in adolescent NSSI appear to be specifically associated with comorbid depressive symptoms rather than self-injurious behavior per se. S2-related inhibitory dysfunction may represent a neurophysiological marker of affective burden and selective executive vulnerability in this population.

## 1 Introduction

Non-suicidal self-injury (NSSI) refers to the repetitive and deliberate destruction of one’s own body tissue without suicidal intent, most commonly through cutting, burning, or hitting oneself (1). Over the past decade, the prevalence of NSSI markedly increased among adolescents, making it a growing public mental health concern (2, 3). Large-scale epidemiological studies in China indicate that the 12-month prevalence of adolescent NSSI reaches approximately 16.9%, with lifetime rates exceeding 25% in high-risk groups such as clinical inpatients and youth with mood disorders (4). NSSI is frequently comorbid with depressive symptoms and has been linked to elevated suicide risk, emotional dysregulation, and impaired psychosocial functioning (5–7).

With growing attention from clinical neuroscience, NSSI is increasingly recognized not solely as a maladaptive coping strategy, but as a neurocognitive phenotype rooted in broader dysfunctions in emotion regulation and cognitive control (8, 9). Specifically, adolescents who engage in NSSI often exhibit deficits in inhibitory control, attentional shifting, and emotion regulation (9). These impairments may amplify reactivity to negative emotional stimuli and environmental stressors, thereby increasing susceptibility to depressive symptoms (10). Understanding the neurophysiological mechanisms that underlie these cognitive-affective vulnerabilities is therefore critical for early identification and targeted intervention.

One candidate mechanism is sensory gating, the brain’s ability to suppress redundant sensory input during early information processing (3). This process is commonly indexed by the P50 component of event-related potentials (ERPs), typically elicited using an auditory paired-click paradigm (11, 12). Reduced P50 suppression reflects impairments in early filtering processes and has been associated with heightened sensory overload, inefficient cognitive resource allocation, and downstream disruption of affective regulation (13). Impairments in P50 sensory gating have been documented in adults with major depressive disorder and other affective conditions, suggesting its potential role as a neurophysiological marker of depression (14).

Despite these findings, relatively few studies have examined P50 sensory gating in adolescents, and its relevance to NSSI remains underexplored, particularly given the high rates of depressive symptoms and affective instability in this population (5, 15). Adolescence is a critical period of neurodevelopment, and disruptions in early sensory processing may have cascading effects on self-regulatory systems (16). Elucidating the nature of P50 gating deficits in adolescents with NSSI, and their association with depressive symptomatology, may offer valuable insights into underlying pathophysiological processes and inform the development of biologically grounded interventions.

The present study aimed to investigate P50 sensory gating function in adolescents with NSSI and to examine the association between P50 suppression and depressive symptoms. We hypothesized that adolescents engaging in NSSI would demonstrate impaired P50 gating compared to controls, and that greater deficits in sensory gating would be associated with more severe depressive symptomatology.

## 2 Materials and methods

### 2.1 Participants

A total of 86 adolescents engaging in NSSI (males/females: 51/35; average age: 15.87 ± 3.75 years) and 50 healthy controls (males/females: 24/26; average age:17.20 ± 3.18 years) were enrolled in this study. Participants were recruited between October 2023 and May 2025 at Tianjin Anding Hospital. This study was approved by the Institutional Ethical Review Board of the hospital. Healthy controls were recruited from the community through advertisement and had no history of psychiatric disorders. The study protocol was approved by the Institutional Ethical Review Board of Tianjin Anding Hospital and conducted in accordance with the Declaration of Helsinki. Written informed consent was obtained from all participants and their legal guardians.

The inclusion criteria of NSSI group were as follows: 1) age 12-18; 2) a documented history of NSSI, defined as deliberate and direct damage to one’s own body tissue without suicidal intent, occurring at least five times within the past 12 months (e.g. cutting, burning, or hitting oneself); 3) ability to complete clinical interviews and EEG recording procedures; 4) sufficient cognitive and communicative capacity for assessment; 5) Han Chinese ethnicity.

The exclusion criteria were as follows: 1) current or lifetime diagnosis of schizophrenia spectrum disorders, bipolar disorder, or other psychotic disorders; 2) the presence of neurological disorders, organic brain diseases, or severe physical illnesses; 3) Substance abuse or dependence (except nicotine); 4) excessive EEG artifacts rendering data unsuitable for analysis.

All participants’ demographic information, including age, sex, years of education, and smoking status, was collected using a uniformly designed questionnaire.

### 2.2 Clinical and psychological assessments

Depressive symptoms were assessed using the HAMD-17 (17, 18). Participants were classified into depressive and non-depressive subgroups using a cutoff score of 8, consistent with prior research identifying clinically meaningful depressive symptomatology in adolescents (19). NSSI adolescents were divided into a comorbid depression group (HAMD-17 score ≥ 8, n = 55) and a non-depression group (HAMD-17 score < 8, n = 31).

Characteristics of NSSI were assessed using standardized questionnaires and structured interviews and standardized self-report instruments derived from validated measures, the Adolescent Non-Suicidal Self-Injury Assessment Questionnaire (20), capturing frequency, methods, and functional aspects of self-injurious behaviors. To further characterize depressive symptoms, the Patient Health Questionnaire-9 (PHQ-9) was administered as a self-report measure (21). Early adverse experiences were evaluated using the Childhood Trauma Questionnaire (CTQ) (22).

Cognitive function was evaluated using the MATRICS Consensus Cognitive Battery (MCCB) (23), which assesses 7 cognitive domains across 10 tasks: processing speed (Trail Making Test Part A, Symbol Coding, and Category Fluency), attention/vigilance (Continuous Performance Test-Identical Pairs, CPT-IP), working memory (Spatial Span and Digit Sequence), verbal learning (Hopkins Verbal Learning Test-Revised, HVLT-R), visual learning (Brief Visuospatial Memory Test-Revised, BVMT-R), problem solving (Neuropsychological Assessment Battery-Mazes), and social cognition (Mayer-Salovey-Caruso Emotional Intelligence Test, MSCEIT). T-scores were standardized and corrected for age and sex using the MCCB scoring program (24). The inter-rater correlation coefficient for the total scores of HAMD-17 and MCCB exceeded 0.8.

### 2.3 P50 sensory gating recording

A brief audiometric screening excluded individuals unable to detect a 1000 Hz, 40 dB tone binaurally. EEG data were acquired using a 64-channel BrainAmp ERP/EP system (Brain Products, Germany) with electrodes placed according to the 10–20 system. Horizontal and vertical electrooculograms were recorded to monitor eye movements and blinks. The nose served as the reference electrode.

P50 sensory gating was assessed using a standard auditory paired-click paradigm, consisting of 150 pairs of clicks (S1-S2 interval: 500ms; stimulus duration: 5ms; rise/fall time:2ms; inter-pair interval:10s) presented binaurally at 90dB (25). Participants were instructed to remain awake, keep their eyes open, and passively ignore the stimuli. P50 sensory gating data were recorded automatically. EEG signals were analog band-pass filtered between 0.1 and 250Hz, with an additional 50Hz notch filter, and digitized at a sampling rate of 500Hz. Electrode impedances were maintained below 10kΩ.

### 2.4 EEG data processing and P50 quantification

Offline EEG data processing was performed using BrainVision Analyzer (Brain Products, Germany). Continuous EEG data were segmented, re-referenced, high-pass filtered at 1Hz, baseline-corrected, and subjected to artifact rejection. Epochs corresponding to the first (S1) and second (S2) stimuli were then averaged separately. For both S1 and S2 conditions, less than 10% of trials were rejected. P50 amplitudes were quantified at the Cz electrode, consistent with established methodological recommendations (26). The P50 response was defined as the most positive deflection occurring within 40–90 ms post-stimulus (27). S1 amplitude was measured peak-to-trough relative to the preceding negative deflection. S2 amplitude was defined using the same criteria, with latency constrained to correspond to the S1 response. Sensory gating indices were calculated using both the S2/S1 ratio and the S1-S2 difference (28), and S1 and S2 amplitudes were included in subsequent analyses. Both raw amplitudes and gating indices were included in statistical analyses.

All waveforms were independently scored by two EEG analysts blinded to clinical status. Discrepancies were resolved through consensus.

### 2.5 Statistical analysis

Statistical analyses were conducted using SPSS 23.0. Data distribution normality was assessed using the Kolmogorov-Smirnov test. Non-normally distributed variables were transformed as appropriate. The S2/S1 gating ratio was log-transformed prior to analysis. Group differences in demographic and clinical variables were evaluated using chi-square tests for categorical variables and one-way ANOVA for continuous variables. When significant differences in P50 indices or cognitive measures were observed, analysis of covariance (ANCOVA) was performed controlling for age, sex, and body mass index (BMI).

Within the NSSI group, Pearson or Spearman correlation analyses (depending on distribution) were used to examine associations between P50 indices and depressive symptom severity (HAMD-17 total score). Multiple linear regression analyses were conducted to assess whether P50 indices independently predicted depressive symptom severity after adjusting for demographic covariates. Logistic regression was additionally performed to identify factors associated with depressive status (HAMD-17 ≥ 8). In addition, subgroup analyses were conducted within the NSSI sample to examine the associations between P50 sensory gating indices and cognitive performance. In each subgroup, correlation analyses were conducted between P50 indices and cognitive measures. Subsequently, multiple linear regression models were constructed within each subgroup to determine whether P50 indices independently predicted cognitive performance after controlling for age, sex and BMI. Standardized β coefficients and adjusted R² values were reported.

To control for multiple comparisons, Bonferroni correction was applied for *post hoc* pairwise comparisons. For the three-group comparisons, the significance threshold was set at *P* < 0.0167 (0.05 divided by 3 comparisons). Corrected *P*-values are indicated in the tables by symbols. All statistical tests were two-tailed, with the level of significance set at *P* < 0.05.

## 3 Results

### 3.1 Demographic characteristics and cognitive performance

As shown in **Table 1**, a total of 136 adolescents were included in the analyses, comprising 50 healthy controls, 55 adolescents with NSSI and comorbid depressive symptoms, and 31 adolescents with NSSI without depressive symptoms. Significant group differences were observed in age (*F* = 4.479, *P* = 0.013). Moreover, *post hoc* comparisons indicated that healthy controls were older than the NSSI without depression group, and the NSSI with depression group was also older than the NSSI without depression group (all *P*s < 0.05).

**Table 1:** Demographic characteristics and MCCB cognitive performance of healthy controls and NSSI participants by depressive symptom subgroup.

Variables	Healthy Controls (n =50)	NSSI (n =86)		*F*	*P* value	Adjusted *F ^ a*	*P* value	Adjusted *F ^ b*	*P* value	Adjusted *F ^ c*	*P* value
		NSSI with Depression (n = 55)	NSSI without Depression (n = 31)								
Sex (Female/Male)	24/26	29/26	22/9		0.117						
Age	17.2 ± 3.18^#^	16.87 ± 3.56^*^	14.9 ± 3.94	4.479	**0.013**						
MCCB total	58.51 ± 9.82^##^	44.35 ± 9.46	47.29 ± 10.56	28.868	**<0.001**	28.629	**<0.001**	55.828	**<0.001**	1.483	0.227
Trail making A	55.02 ± 8.64^##^	46.24 ± 6.62	47.9 ± 7.73	18.496	**<0.001**	17.837	**<0.001**	34.529	**<0.001**	0.979	0.325
Symbol coding	54.35 ± 9.46^##^	39.56 ± 11.03	42.84 ± 10.36	28.472	**<0.001**	30.969	**<0.001**	61.424	**<0.001**	0.628	0.43
Verbal learning	57.49 ± 8.44^##^	47.02 ± 10.47	50.06 ± 9.71	16.097	**<0.001**	16.21	**<0.001**	30.957	**<0.001**	1.231	0.27
Spatial span	56.6 ± 11.52^##^	45.25 ± 11.67	46.87 ± 13.03	13.034	**<0.001**	13.417	**<0.001**	26.901	**<0.001**	0.105	0.747
Mazes	58.66 ± 8.63^##^	46.51 ± 10.02	50 ± 11.58	20.277	**<0.001**	20.17	**<0.001**	37.449	**<0.001**	2.301	0.133
Visual learning	57.74 ± 8.56^##^	46.47 ± 9.23	45.81 ± 9.82	25.178	**<0.001**	22.338	**<0.001**	44.846	**<0.001**	0.324	0.571
Category Fluency	53.18 ± 8.81	51.05 ± 9.18	50.06 ± 9.77	1.266	0.285	1.045	0.355	1.962	0.164	0.048	0.827
Social cognition	49.48 ± 8.05	48.84 ± 11.66	50.74 ± 11.21	0.336	0.716	0.263	0.769	0.002	0.962	0.446	0.506
CPT-IP	52.38 ± 7.61^##^	41.47 ± 9.7	46.06 ± 9.7	19.35	**<0.001**	19.623	**<0.001**	34.794	**<0.001**	3.286	0.074
Digital Sequence	55.63 ± 9.94^##^	48.04 ± 10.77	48.39 ± 9.2	8.598	**<0.001**	10.416	**<0.001**	20.759	**<0.001**	0.128	0.722

Significant differences are highlighted in bold.

Data presented as mean ± standard deviation.

Bonferroni correction was applied for *post hoc* pairwise comparisons. For the three-group comparisons, the significance threshold was set at *P* < 0.0167 (0.05/3).

Hashtag indicates significance of the comparisons between healthy controls and NSSI participants (both with and without depressive symptoms), ^##^*P* < 0.01, ^#^*P* < 0.05.

Asterisk indicates significance of the comparisons between NSSI with Depression and NSSI without Depression subgroups, ^*^P < 0.05.

Abbreviations: NSSI, non-suicidal self-injury; MCCB, Measurement and Treatment Research to Improve Cognition in Schizophrenia (MATRICS) Consensus Cognitive Battery; CPI - IP, Continuous Performance Test.

^a Comparisons between three groups adjusted by age, sex.

^b Comparisons between healthy controls and NSSI group adjusted by age, sex.

^c Comparisons between depression and non-depression subgroups adjusted by age, sex.

Significant group effects were observed across the majority of MCCB domains, including the total MCCB score, Trail making A (Trail Making Test Part A), Symbol coding, Verbal learning, Spatial span, Mazes, Visual learning, CPT-IP and Digital sequence (all *P*s < 0.01). No significant group differences were detected in Category Fluency or Social Cognition.

*Post hoc* analyses revealed that healthy controls demonstrated significantly higher cognitive performance compared with both NSSI subgroups across the significant domains. However, no significant differences were observed between the NSSI with depression and NSSI without depression groups on the MCCB composite or any domain-specific scores. All reported differences remained significant after Bonferroni correction.

### 3.2 P50 suppression and parameters

**Figure 1:**
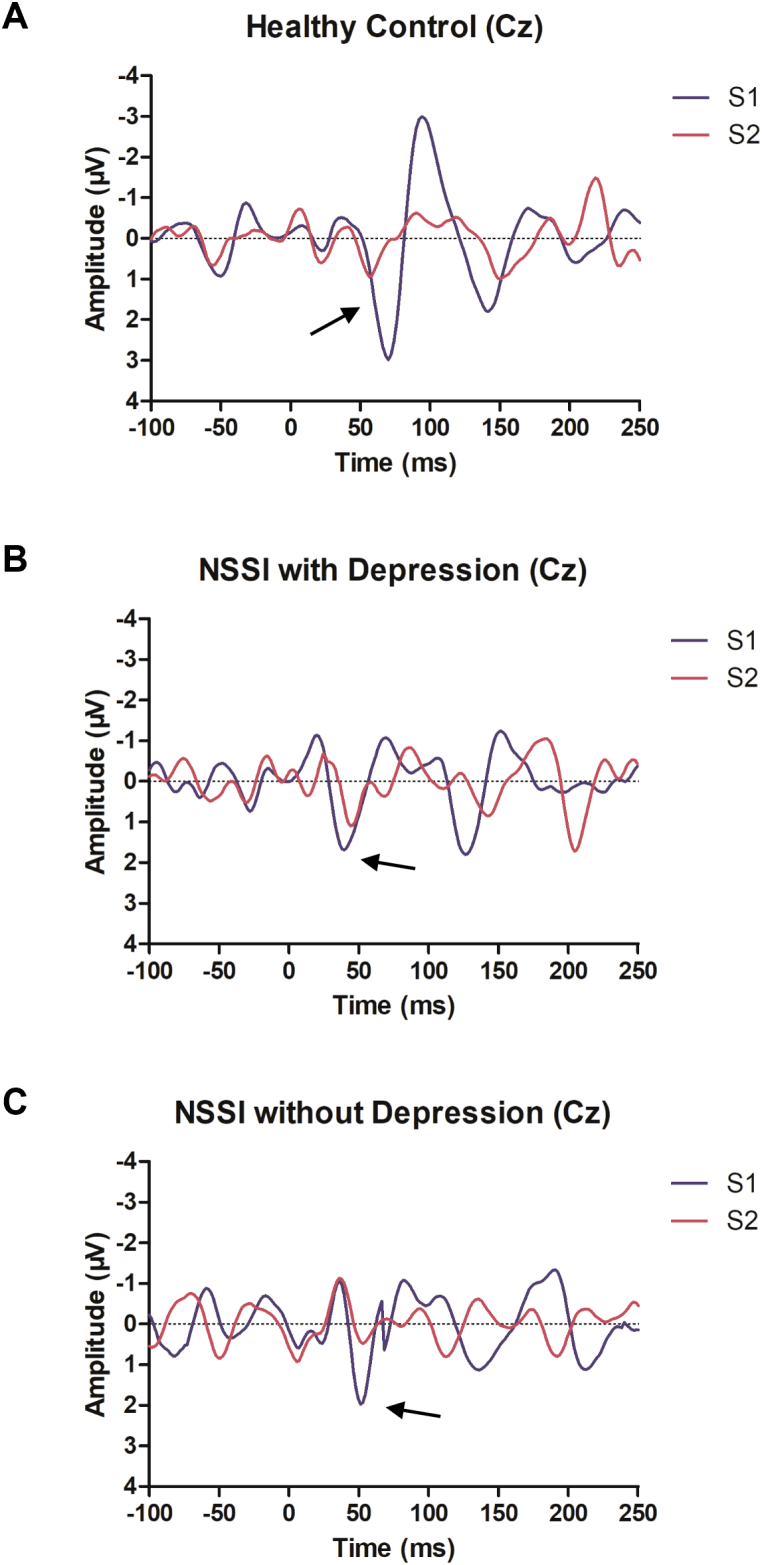
Average data of the SG-P50 waveforms at the Cz site for healthy controls **(A)**, NSSI with depression **(B)** and NSSI without depression **(C)**, respectively. Red arrow indicates the peak of P50 wave of S2. SG, sensory gating; NSSI, non-suicidal self-injury.

Grand-average P50 waveforms recorded at Cz are presented in **Figures 1A–C**, and detailed parameters are summarized in **Table 2**.

**Table 2:** Comparisons of P50 measures and HAMD-17 scores among healthy controls and NSSI participants with and without depressive symptoms.

Variables	Healthy Controls (n =50)	NSSI (n =86)		*F*	*P* value	Adjusted *F ^ a*	*P* value	Adjusted *F ^ b*	*P* value	Adjusted *F ^ c*	*P* value
		NSSI with Depression (n = 55)	NSSI without Depression (n = 31)								
S1 lat (mA)	56.44 ± 7.65	61.13 ± 7.68^##^	60.45 ± 6.9	5.633	**0.004**	4.962	**0.008**	9.367	**0.003**	0.390	0.534
S1 amp (uV)	3.36 ± 1.69	3.05 ± 1.76	3.12 ± 2.06	0.412	0.663	0.565	0.570	1.133	0.289	0.061	0.806
S2 lat (mA)	56.52 ± 11.14	58.29 ± 10.3	60 ± 7.85	1.159	0.317	0.888	0.414	1.407	0.238	1.167	0.283
S2 amp (uV)	1.12 ± 1.14	1.55 ± 1.15	0.93 ± 0.72	3.925	**0.022**	3.392	**0.037**	1.669	0.199	5.279	**0.024**
S2 amp/S1 amp ratio (%)	35.3 ± 29.35	63.89 ± 58.41^##^	46.74 ± 45.2	5.018	**0.008**	5.172	**0.007**	8.688	**0.004**	0.976	0.326
S1 amp-S2 amp (uV)	2.24 ± 1.6	1.52 ± 1.84	2.2 ± 2.09	2.444	0.091	2.338	0.100	3.230	0.075	0.891	0.348

Significant differences are highlighted in bold.

Data presented as mean ± standard deviation.

Bonferroni correction was applied for *post hoc* pairwise comparisons. For the three-group comparisons, the significance threshold was set at *P* < 0.0167 (0.05/3).

Hashtag indicates significance of the comparisons between healthy controls and NSSI participants (both with and without depressive symptoms), ^##^*P* < 0.01.

NSSI, non-suicidal self-injury; S1, conditioning stimulus; S2, testing stimulus; S1 lat, latency of the P50 component to S1; S2 lat, latency to S2; S1 amp, amplitude to S1; S2 amp, amplitude to S2; S2 amp/S1 amp ratio, P50 suppression ratio; S1 amp-S2 amp, difference between S1 and S2 amplitudes.

^a Comparisons between three groups adjusted by age, sex.

^b Comparisons between healthy controls and NSSI group adjusted by age, sex.

^c Comparisons between depression and non-depression subgroups adjusted by age, sex.

Significant group differences were observed for S2 amplitude (*F* = 3.392, *P* = 0.037), S2/S1 ratio (*F* = 5.172, *P* = 0.007), and S1 latency (*F* = 4.962, *P* = 0.008). *Post hoc* comparisons demonstrated that adolescents with NSSI and comorbid depression exhibited significantly elevated S2/S1 ratios and prolonged S2 latency compared with healthy controls (*P*s < 0.01). No significant differences were observed between the NSSI without depression group and healthy controls.

After controlling for age and sex using ANCOVA, the difference in S2 amplitude between the NSSI with depression and NSSI without depression subgroups remained significant (*F* = 5.279, *P* = 0.024), indicating that sensory gating impairment was primarily driven by the depression comorbidity within the NSSI population.

### 3.3 Relationships between P50 parameters, and depressive symptoms

**Figure 2:**
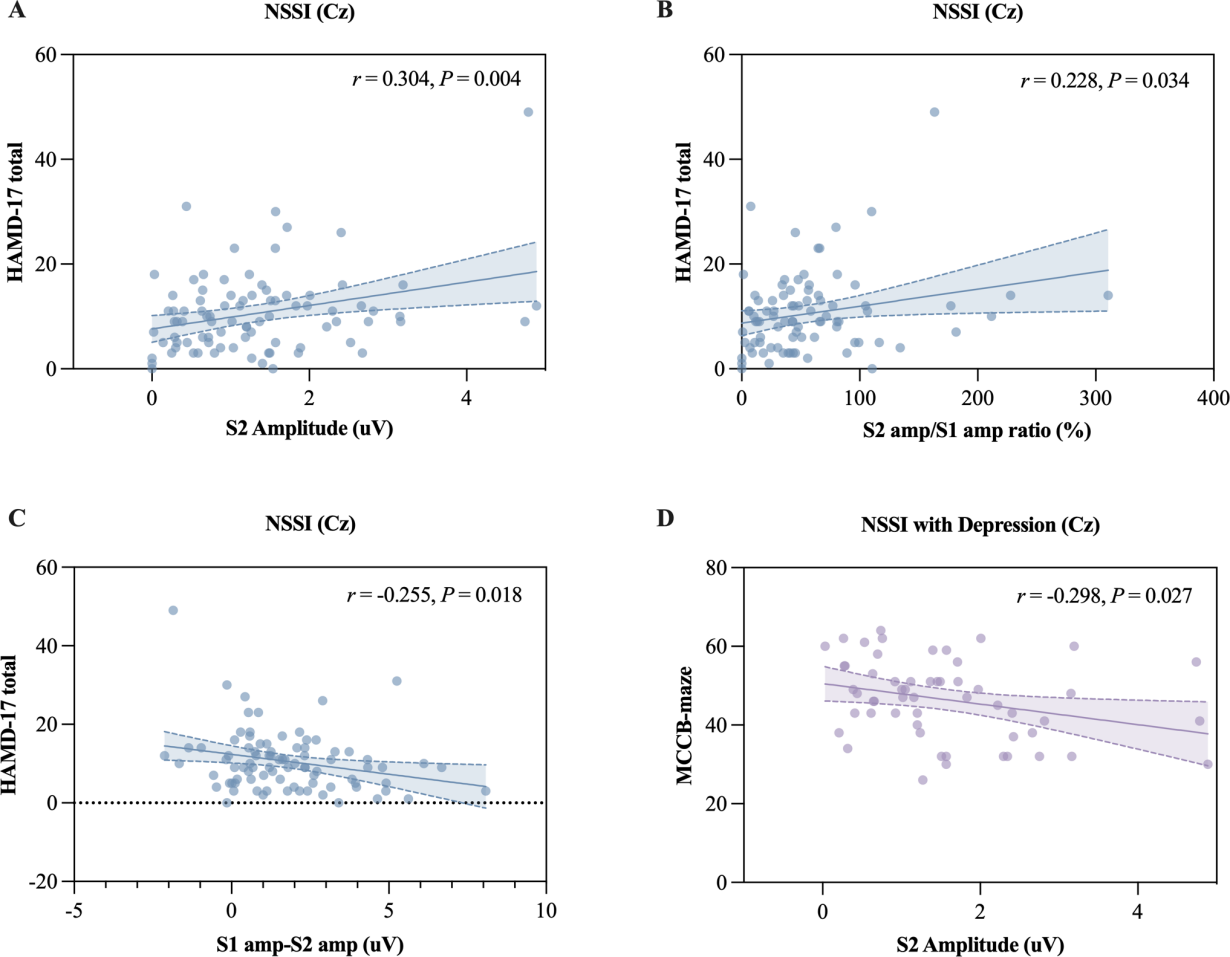
Correlation analysis revealed significant associations between SG-P50 indices and HAMD-17 total scores in NSSI participants: **(A)** S2 amplitude and HAMD-17 total score in NSSI group (*r* = 0.304, *P* = 0.005), **(B)** S2 amp/S1 amp ratio and HAMD-17 total score in NSSI group (*r* = 0.229, *P* = 0.034), **(C)** S1 amp-S2 amp and HAMD-17 total score in NSSI group (*r* = -0.255, *P* = 0.018), **(D)** S2 amplitude and MCCB-Maze in NSSI with depression subgroup (*r* = -0.298, *P* = 0.027). SG, sensory gating; NSSI, non-suicidal self-injury; HAMD-17, Hamilton Depression Rating Scale-17 items; S1, conditioning stimulus; S2, testing stimulus; S1 amp, amplitude to S1; S2 amp, amplitude to S2; S2 amp/S1 amp ratio, P50 suppression ratio; S1 amp-S2 amp, difference between S1 and S2 amplitudes.

Within the overall NSSI group, depressive symptom severity (HAMD-17 total score) was significantly correlated with S2 amplitude (*r* = 0.304, *P* = 0.004), S2/S1 ratio (*r* = 0.228, *P* = 0.034), and S1-S2 difference (*r* = -0.255, *P* = 0.018) (**Figures 2A–C**). As presented in **Table 3**, stepwise regression results show that S2 amplitude was an independent predictor of HAMD-17 score (*β* = 0.264, *t* = 2.671, *P* = 0.009). No other P50 parameter remained significant in the adjusted model.

**Table 3:** Stepwise linear regression analyses of P50 measures significantly predicting HAMD-17 and MCCB cognitive scores in healthy controls and NSSI participants with and without depressive symptoms.

Group	DV	Variables	B	SE	β	T	*P*-value	95% CI for B	Adjusted R²	VIF
NSSI	HAMD total								0.18	
		Sex	-5.17	1.55	-0.33	-3.335	**0.001**	-8.254 - -2.086		1.015
		S2 amp (uV)	1.944	0.728	0.264	2.671	**0.009**	0.496 - 3.393		1.015
	Visual learning								0.131	
		Age	0.852	0.253	0.345	3.366	**0.001**	0.349 - 1.356		1.026
		S2 lat (mA)	-0.24	0.101	-0.242	-2.366	**0.02**	-0.442 - -0.038		1.026
NSSI with Depression	Maze								0.072	
		S2 amp (uV)	-2.609	1.147	-0.298	-2.274	**0.027**	-4.91 - -0.308		1
	Visual learning								0.144	
		Age	0.984	0.334	0.379	2.946	**0.005**	0.314 - 1.654		1.046
		S2 lat (mA)	-0.245	0.115	-0.274	-2.128	**0.038**	-0.477 - -0.014		1.046
	Digital Sequence								0.119	
		Sex	6.045	2.737	0.283	2.209	**0.032**	0.553 - 11.537		1.005
		S1 lat (mA)	0.403	0.18	0.287	2.244	**0.029**	0.043 - 0.764		1.005

Significant differences are highlighted in bold.

Data presented as mean ± standard deviation.

NSSI, non-suicidal self-injury; DV, dependent variable; S1, conditioning stimulus; S2, testing stimulus; S1 lat, latency of the P50 component to S1; S2 lat, latency to S2; S2 amp, amplitude to S2.

### 3.4 Associations between P50 parameters and cognitive performance

In the overall NSSI group, S2 latency was not significantly associated with MCCB Visual Learning scores in correlation analysis (*r* = -0.188, *P* = 0.084). However, after adjusting for age and sex in regression analysis, S2 latency emerged as a significant independent predictor of Visual Learning performance (*β* = -0.242, *t* = -2.366, *P* = 0.020).

Subgroup analyses revealed a more specific pattern. In the NSSI with depression subgroup, S2 amplitude was significantly correlated with Maze performance (*r* = -0.298, *P* = 0.027) (**Figure 2D**). Regression analysis confirmed that S2 amplitude independently predicted Maze scores after controlling for demographic variables. No significant associations between P50 parameters and cognitive performance were observed in the NSSI without depression subgroup.

## 4 Discussion

The present study investigated P50 sensory gating in adolescents with NSSI and revealed that gating abnormalities were primarily confined to those with comorbid depressive symptoms. Specifically, adolescents in the NSSI with depression subgroup exhibited significantly impaired P50 suppression compared with both healthy controls and NSSI participants without depression. Within the overall NSSI sample, S2 amplitude was independently associated with depressive symptom severity, suggesting that deficient inhibitory suppression of the second stimulus response may contribute to affective burden in this population. Moreover, S2 amplitude was selectively related to problem-solving performance (Maze task) in the NSSI with depression subgroup, whereas no broad cognitive differences were observed between NSSI subgroups.

P50 sensory gating reflects pre-attentive inhibitory control mediated by a distributed neural circuit involving the hippocampus, prefrontal cortex, thalamus, and cholinergic projections (27, 29). Reduced suppression of the second stimulus (S2) is generally interpreted as a failure of inhibitory modulation rather than diminished primary sensory registration (30, 31). Heightened S2 amplitude therefore likely reflects inefficient filtering of redundant information, resulting in increased cortical noise and reduced signal-to-noise ratio in downstream processing streams (30, 32). In the context of depression, heightened S2 responses may reflect compromised top-down modulation of sensory input, potentially amplifying negative environmental cues and sustaining maladaptive affective states (14, 33). The independent association between S2 amplitude and depressive severity is consistent with the hypothesis that early-stage inhibitory dysfunction may contributes to affective dysregulation rather than being a mere epiphenomenon of self-injurious behavior.

Importantly, the absence of significant P50 abnormalities in the NSSI without depression subgroup suggests that sensory gating deficits are not a universal characteristic of self-injury (34). Instead, they may index a depression-related vulnerability dimension embedded within the NSSI phenotype (35). This pattern aligns with dimensional models of psychopathology, which propose that early sensory processing abnormalities may serve as transdiagnostic vulnerability markers linked to affective burden rather than to categorical diagnoses (36, 37). Notably, S2 amplitude, rather than the S2/S1 ratio, emerged as the most robust correlate of depressive severity. This finding suggests that the critical dysfunction lies in excessive residual response to the second stimulus, potentially reflecting compromised inhibitory modulation of repeated input (25). Such S2-specific alterations may provide a more direct neurophysiological index of inhibitory failure in adolescent affective dysregulation. The subgroup-specific association between S2 amplitude and Maze performance raises the possibility that depressive symptomatology may influence the link between early sensory inhibition and executive functioning. However, as interaction effects were not formally tested in the current study, this interpretation remains preliminary. Future studies should employ moderation analyses to determine whether depressive status significantly modifies the relationship between sensory gating and executive control.

The selective association between S2 amplitude and Maze task performance in the NSSI with depression subgroup provides additional support for a cascading model of dysfunction. The Maze task primarily indexes executive planning and problem-solving abilities, which depend on efficient allocation of cognitive resources and intact prefrontal control mechanisms (23). Impaired early sensory inhibition may elevate baseline neural noise, thereby reducing processing efficiency in executive networks, particularly under conditions of affective burden (38). The absence of broad cognitive associations across domains suggests that sensory gating deficits do not produce generalized cognitive impairment but instead interact selectively with executive systems when depressive symptomatology is present. This pattern is compatible with neurodevelopmental vulnerability models proposing that disruptions in early inhibitory mechanisms during adolescence may interact with affective dysregulation to compromise higher-order control processes (39, 40).

Several limitations should be considered. First, the cross-sectional design limits causal inference regarding whether P50 abnormalities precede or result from depressive symptomatology. Second, although subgroup analyses yielded meaningful effects, sample size constraints may limit detection of subtler cognitive associations. Third, while the cutoff of HAMD 17 ≥ 8 has been used in previous studies, it may have captured adolescents with relatively mild depressive symptoms. Future studies employing structured diagnostic interviews or higher cutoffs are needed to confirm the specificity of our findings. Fourth, although nicotine abstinence was controlled prior to EEG recording, psychotropic medication exposure was not systematically controlled in the current study. Medication effects should be addressed in future studies using medication-naïve samples or more rigorous pharmacological control. Given the broad adolescent age range included in the present study, and the known developmental maturation of inhibitory processes, age-related neurodevelopmental variation may have partially influenced P50 parameters despite statistical adjustment. Longitudinal designs will be necessary to disentangle developmental effects from symptom-related alterations. Longitudinal and multimodal studies integrating source localization and circuit-level imaging are needed to determine whether S2-related gating deficits may represent a stable neurobiological vulnerability marker or a state-dependent feature of depressive burden in adolescent NSSI.

## 5 Conclusion

In summary, the present study demonstrates that P50 sensory gating impairment in adolescents with NSSI is primarily evident in those with comorbid depressive symptoms. Increased S2 amplitude was independently associated with depressive severity within the NSSI group and, specifically in the NSSI with depression subgroup, was also linked to impaired problem-solving performance. These findings suggest that early sensory inhibition deficits may contribute to affective vulnerability and selective executive dysfunction in a depression-related neurobiological subtype of adolescent NSSI. Identifying S2-related sensory gating abnormalities may therefore help refine neurophysiological characterization and risk stratification within this clinically heterogeneous population.

## Data Availability

The original contributions presented in the study are included in the article/supplementary material. Further inquiries can be directed to the corresponding author.
